# Adaptive design of a clinical decision support tool: What the impact on utilization rates means for future CDS research

**DOI:** 10.1177/2055207619827716

**Published:** 2019-02-06

**Authors:** Devin Mann, Rachel Hess, Thomas McGinn, Rebecca Mishuris, Sara Chokshi, Lauren McCullagh, Paul D. Smith, Joseph Palmisano, Safiya Richardson, David A. Feldstein

**Affiliations:** 1 Department of Population Health, New York University School of Medicine, United States of America; 2 Department of Population Sciences, University of Utah School of Medicine, United States of America; 3 Division of General Internal Medicine, Hofstra Northwell School of Medicine, United States of America; 4 Department of Medicine, Boston University, United States of America; 5 Department of Medicine, University of Wisconsin School of Medicine and Public Health, United States of America

**Keywords:** User-centered design, clinical decision support, usability, health informatics, provider adoption

## Abstract

**OBJECTIVE:**

We employed an agile, user-centered approach to the design of a clinical decision support tool in our prior integrated clinical prediction rule study, which achieved high adoption rates. To understand if applying this user-centered process to adapt clinical decision support tools is effective in improving the use of clinical prediction rules, we examined utilization rates of a clinical decision support tool adapted from the original integrated clinical prediction rule study tool to determine if applying this user-centered process to design yields enhanced utilization rates similar to the integrated clinical prediction rule study.

**MATERIALS & METHODS:** We conducted pre-deployment usability testing and semi-structured group interviews at 6 months post-deployment with 75 providers at 14 intervention clinics across the two sites to collect user feedback. Qualitative data analysis is bifurcated into immediate and delayed stages; we reported on immediate-stage findings from real-time field notes used to generate a set of rapid, pragmatic recommendations for iterative refinement. Monthly utilization rates were calculated and examined over 12 months.

**RESULTS:**

We hypothesized a well-validated, user-centered clinical decision support tool would lead to relatively high adoption rates. Then 6 months post-deployment, integrated clinical prediction rule study tool utilization rates were substantially lower than anticipated based on the original integrated clinical prediction rule study trial (68%) at 17% (Health System A) and 5% (Health System B). User feedback at 6 months resulted in recommendations for tool refinement, which were incorporated when possible into tool design; however, utilization rates at 12 months post-deployment remained low at 14% and 4% respectively.

**DISCUSSION:**

Although valuable, findings demonstrate the limitations of a user-centered approach given the complexity of clinical decision support.

**CONCLUSION:**

Strategies for addressing persistent external factors impacting clinical decision support adoption should be considered in addition to the user-centered design and implementation of clinical decision support.

## Background and Significance

Clinical decision support (CDS) tools have been at the forefront of digital health solutions for more than 10 years.^1,2^ The implementation of the Health Information Technology for Economic and Clinical Health and Affordable Care Acts sets the stage for widespread testing and adoption of CDS tools to promote optimal care delivery. Yet, these objectives have been constrained by well-documented barriers to CDS effectiveness, including poor usability, alert fatigue, and low utilization.^3–5^ Together, these barriers have impeded the impact of CDS on health outcomes, quality of care, and cost reductions.^6–8^

### User-centered CDS

To address these barriers, CDS adoption studies have increasingly used an agile, user-centered design (UCD) approach with a focus on human-computer interaction (HCI).^9^ In these approaches, CDS tools are created in collaboration with users to best reflect needed content, workflow, and ease of use.^10,11^ Commonly used in digital marketing and online commerce, user-centered design principles are increasingly used by the healthcare industry to increase usability, acceptability, and effectiveness of healthcare information technologies.^12,13^ In a systematic review comparing models of adoption of CDS, the most significant factors driving adoption was a system’s ability to be dynamic, launch “multiple assumptions,” and incorporate “new information in response to changing circumstances,” all factors heavily dependent on an explicit understanding of system end users.^14^

**Figure 1.**
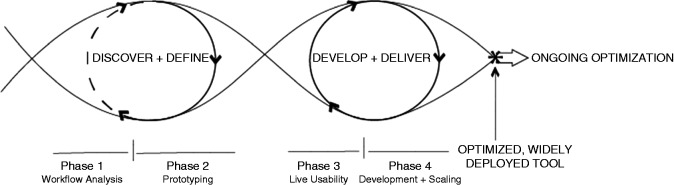
Process model for user-centered digital development.

Figure 1 illustrates our user-centered process model for digital design used in this and previous CDS development projects.^15^ The four-phase model, executed by a team of multidisciplinary stakeholders, uses a process of discovery paired with product definition and development phases characterized by rapid cycle agile testing—an implementation involving wireframing, workflow analysis, and usability testing—to develop and update tool design based on feedback regarding user needs and shifting contexts.^16,17^ Importantly, the model takes into consideration the CDS module lifecycle, including the need to consider adaptations or further “optimization” as additional user needs are identified or changes in practice or technology, such as electronic health record (EHR) functionality, are encountered.

Our previous study (referred to as integrated clinical prediction rule 1 (iCPR1)) employed this UCD framework to develop an iCPR CDS tool for reducing antibiotic prescribing for upper respiratory infections.^18^ The design and workflow of the tool was created through iterative cycles of design, function, and usability testing, and content creation and revision with stakeholders; this resulted in a CDS tool with an adoption rate five times greater than previous reports.^19,20^ In nearly two-thirds of opportunities, clinicians used the offered iCPR1 tool, an antibiotic evidence-based CDS pathway, in more than half of all iCPR1 encounters; they went on to use the associated bundled order set (a pre-specified set of medications, tests, and documentation). This represents a substantial improvement from earlier studies of CDS tools for acute upper respiratory infection, which demonstrated utilization rates as low as 6%.^21^ The success of the original iCPR1 tool points to the strengths of the agile, user-centered approach to CDS tool design and highlights the value of our tailored application of this industry practice for academic research.^22–24^

### Adapted iCPR study to date

Our objectives for the adapted iCPR project (iCPR2) were to apply this approach to adapt and scale up the implementation of the iCPR CDS tool to a diverse group of 33 primary care clinics across multiple institutions. To understand if applying a user-centered process to adapt CDS tools is effective in improving the use of clinical prediction rules, we assessed resulting utilization rates, and ultimately, the tool’s impact on antibiotic prescription rates for acute respiratory infection in two academic health systems. Prior publications from this project outline the initial user-centered tool design process including initial tool development based on feedback from assessment-focused key informant interviews with providers, clinic managers, and medical assistants at each site.^18,19,25,26^ Findings highlight an understanding of general clinic workflows, specific workflows, policies, and practices around rapid strep and chest x-ray testing, and clinic organizational structure as related to implementation process and tool requirements.^25^ Near-live and live usability testing was conducted, and the iCPR2 tool iteratively revised accordingly, as reported in previous publications.^18,19,26–28^

## Objectives

The objective of this study was to examine utilization rates of the adapted tool (iCPR2) across sites over 12 months post-deployment to determine if applying this user-centered process to the design of the tool yields enhanced utilization rates similar to that in iCPR1.

## Methods

### Study design

As part of a large, randomized controlled trial involving over 40,000 visits in which we adapt and scale up the implementation of the iCPR1 tool to diverse clinics in two academic health systems, we collected qualitative user feedback during tool development, and quantitative and quantitative data on tool use over the first 12 months of implementation. Utilization data were reviewed biweekly for 12 months post-deployment to assess ongoing engagement with the tools by site and to determine the need for and direction of further user feedback, data collection, and tool iteration, with the goal of identifying barriers and facilitators to use as indicated by differences in utilization rates post-deployment.

### Data collection and analysis

#### Usability

Qualitative usability data were collected *pre-deployment* using: 1) key informant interviews with CDS and clinical content experts and local clinical leaders, 2) think-aloud, 3) near-live, and 4) live usability methodologies to gather information for initial tool development and iteration; the use of these methodologies in collecting usability data for the purposes of adaptive design is described in detail in our previous publications and summarized below.^18,19,27,28^

The initial prototype of the iCPR2 tool was subjected to multiple rounds of usability testing in increasingly realistic clinical simulations. During these usability tests, providers (*n*=12) were presented with simulated cases and asked to “think-aloud” by verbalizing thought processes as they interacted with the prototype iCPR2 tool to examine usability aspects of the tool.

Think-aloud sessions were followed by “near-live” testing with the same prototype in which provider volunteers were asked to interact with a simulated patient to collect data on how the tool fit (or did not fit) with provider workflows. Observational “live” usability was conducted with three providers using the tool in six real clinical encounters to assess previously unidentified barriers to tool use. *Post-deployment* usability data were collected via semi-structured group interviews with providers across a convenience sample of the 33 intervention clinics (*n*=14) to collect qualitative user feedback 6 months post-deployment. All providers (physicians, nurses, nurse practitioners, and medical assistants) who may have been exposed to the iCPR2 tool in practice at intervention sites were invited via clinic administrators to participate in a 30–45 minute group interview session in which they were asked questions related to usability aspects of the tool (see Table 1 for sample questions from the interview guide).

**Table 1.** Post-deployment usability feedback interview guide excerpt.

**Interview question**	**Usability theme**
How have the results of the tool, including the smartsets, been useful or not when providing care to your patients?	**Utility**
Does the tool trigger when you expect it to?	**Workflow**
Is the tool easy to use?	**Ease of use**
How has your time with patients been affected by use of the tool?	**Burden**

Full screen capture and audio was recorded for each think-aloud, near-live, and live-usability session using *Morae*® and *Camtasia*® software. Post-deployment group interviews were recorded with detailed field notes and then summarized. Qualitative usability data analysis is bifurcated into immediate and delayed stages; this paper reports immediate stage findings aimed at generating rapid, pragmatic recommendations for iterative refinement as they apply to design decisions and tool utilization. Participant comments were placed into a priori (according to key usability principles) as well as inductively derived coding categories, and analyzed for generalizable themes to translate into tool design recommendations. The delayed phase of analysis features deeper, systematic thematic analysis of transcribed field notes to identify generalizable insights for CDS.

#### Utilization

To determine the rate and variability in utilization of the iCPR2 tool across settings, monthly utilization rates (as measured by calculator completion) at each participating health system were calculated and compared at 6 and 12 months post-deployment to determine if utilization increased with tool iteration. To accomplish this, weekly reports were generated on the utilization of each step of the iCPR2 tool pathways for each intervention site. These reports identified rates of tool triggering by the relevant chief complaint and actions taken (or not taken) by the providers (in aggregate and by clinical site).

### Study sites

The study was conducted at primary care clinics associated with two large academic health systems: one Midwestern (Health System A) and the other in the Intermountain United States (Health System B). All general internal medicine (GIM) and family medicine (FM) primary care clinics at the two institutions were invited to participate. A total of 33 individual clinics (12 GIM clinics, 16 FM clinics, and five combined clinics) participated in the study. Each site used the same EHR system (Epic Systems, Verona, WI) and its native functionality (i.e., meaning no custom software development in addition to what is standardly available in the EHR was used) to develop the iCPR tools in their EHR. Each site was supported by an information technology department that adapted and tested the components of the iCPR tool before deployment.

### The tool: iCPR2

The iCPR2 tool and workflow were adapted from those developed in our original iCPR study, as outlined above.^19^ In the pre-deployment phase of the current study, the iCPR2 design was developed by an interdisciplinary team of experts in primary care, usability, and clinical informatics at each institution (see Feldstein et al. for details on the design process).^25^

**Figure 2.**
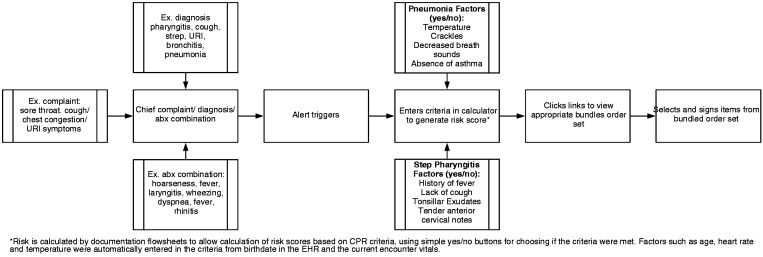
Adapted integrated clinical prediction rule 1 (iCPR2) project tool and provider workflow.

The initial iteration of the new iCPR2 tool was triggered by relevant activity in any of the following EHR fields: chief complaint, diagnosis, or diagnosis along with antibiotic ordering. When triggered, the clinician is presented with an alert offering the iCPR2 tool upon opening the chart. If the alert is accepted (versus dismissed or ignored), the clinician is taken to a screen with a list of clinical questions, each of which contributes to a total risk score (see calculator description below with depiction in Figure 2), based on the triggering information. Temperature and heart rate are automatically populated, as applicable, based on vital signs logged by the medical assistant. After completing the tool, clinicians are then offered a pre-populated order set based on the calculated risk (Figure 2).

## Results

### Pre-deployment results: User feedback and subsequent adaptation of iCPR tool

**Figure 3.**
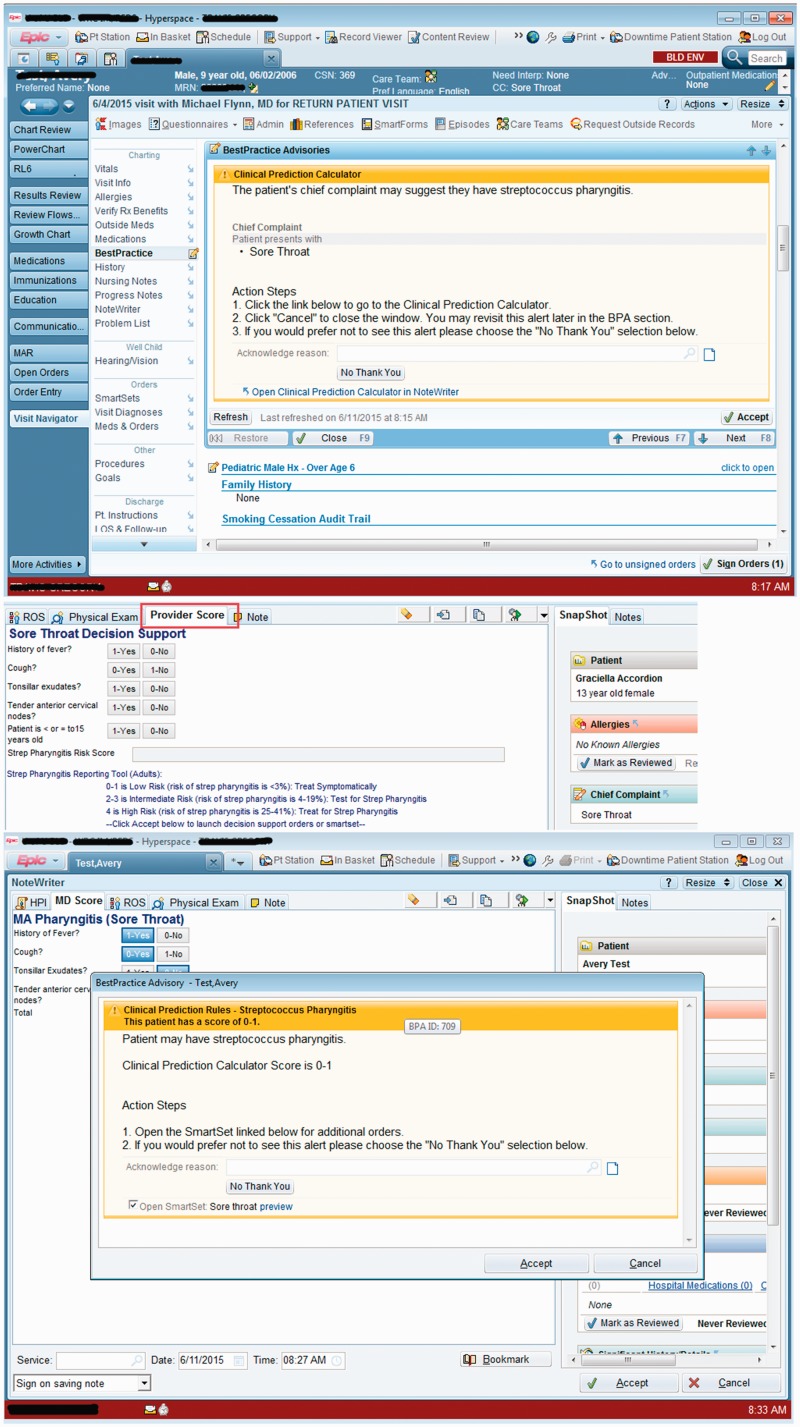
Health System B’s visually integrated non-interruptive alert. © 2017 Epic Systems Corporation. Used with permission.

The iCPR2 tool was modified based on user feedback in the prototype phase at each site to reflect the workflow and other key clinical process differences between the sites. For example, across Health System B, primary care providers heavily leveraged an EHR-assisted documentation pathway in which the medical assistants typically completed the chief complaint field and initiated a structured history of the present illness. Results of near-live usability testing indicated an opportunity to leverage this default workflow at Health System B by modifying the tool so that medical assistants gathered pieces of the history or physical exam that were relevant to iCPR2 and prepopulated the respective fields in the provider’s view of the tool. As a result, from the provider perspective, the iCPR2 tool was embedded into the usual collaborative documentation workflow with an additional EHR section with the standard view representing the partially completed iCPR2 strep or pneumonia risk calculator tool (see Figure 3).

**Figure 4.**
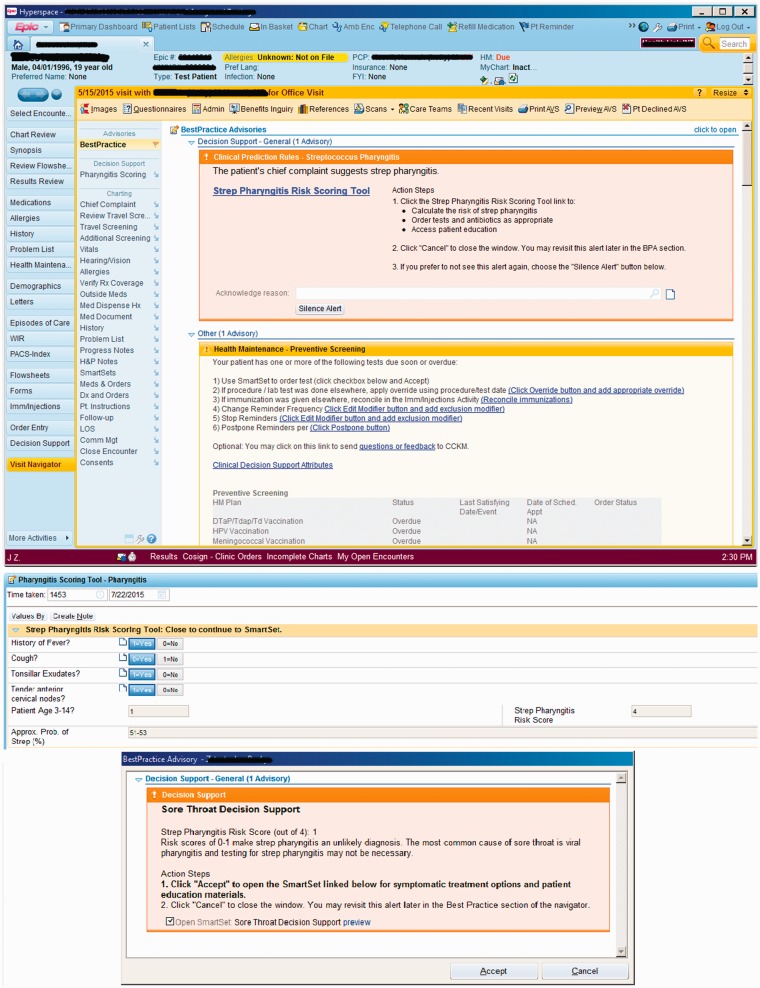
Health System A’s highlighted non-interruptive alert. © 2017 Epic Systems Corporation. Used with permission.

In contrast, the Health System A-affiliated clinical sites do not use structured documentation and preferred a version of iCPR2 that adhered more closely to the original provider-initiated iCPR workflow. After a relevant chief complaint was entered, a visibly highlighted non-interruptive alert was presented to the provider that encouraged engagement with the iCPR2 tool (Figure 4). Further information on design and the usability testing during development of the tools can be found in our three previous papers.^18,25,26^

Table 2 features usability design themes identified from qualitative usability data collected in the pre-deployment phase of the user-centered design process (see Methods for description of analysis approach) matched with resulting modifications made in tool design prior to iCPR2 tool deployment.

In response to clinician feedback related to concerns regarding the negative effects on workflow and increased burden of potentially irrelevant tool triggers and alert fatigue, less-specific chief complaint triggers of cough, upper respiratory infection, and sore throat were chosen instead of a previously used (in iCPR) longer list of more specific but potentially mismatched chief complaints, ultimately prioritizing alert sensitivity over specificity. To balance out the low specificity triggers and minimize clinician frustration and alert fatigue, we chose to use non-interruptive alerts (not requiring user action to resolve the alert in order to continue with work) for the initial iCPR2 chief complaint-based trigger.^29,30^ We also explored EHR functionality that could allow for non-interruptive alerts for the triggers based on the visit diagnosis triggers, however, no mechanisms exist within the EHR to allow for this, so we initially opted for interruptive alerts if iCPR was triggered by a relevant diagnosis or diagnosis plus antibiotic prescription.

**Figure 5.**
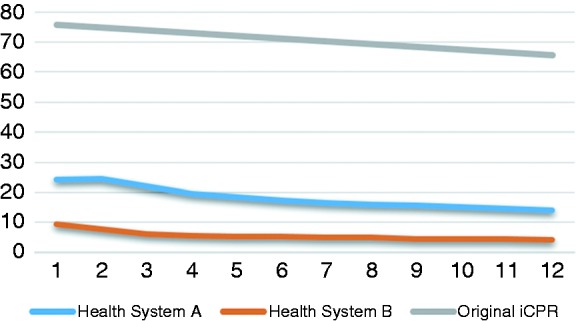
Utilization of integrated clinical prediction rule 1 (iCPR) and adapted iCPR1 (iCPR2) (both conditions).

**Table 2.** Pre-deployment user feedback excerpts and tool modifications per usability theme.

Usability driver/issue	User excerpt	Design decision/modification
Prepopulated data fields leverage natural workflows/minimize workload	“I liked the fact that it actually obtains and pulls in the clinical information that’s discrete, that’s available, such as the heart rate and the temperature.”	Pieces of patient history gathered by medical assistant prepopulate in providers’ view per site natural workflow.
Interruptive alerts disruptive to natural workflow	"I much prefer to have stuff in the background that doesn’t force me to have hard stops… There may be a whole series of other things I’m dealing with."	Created static alert able to be silenced permanently by provider.
Lack of visibility	“But the score is way over on the right so I didn’t actually notice what the score was.”	Alert moved to left side.
Evidence up-to-date and specialty specific	“I don’t even know that I would give kids cephalexin because again it tastes horrible.”	Medications in smartsets aligned with organizational recommendations

### Post-deployment phase: Utilization rates and user feedback

Figure 5 illustrates the overall rates of the iCPR2 tool utilization at each site for both strep and pneumonia combined as compared with utilization rates (as measured by calculator completion rates) seen in the iCPR1 study over the first year of implementation.

By 6 months post-deployment, it had become apparent that iCPR2 tool utilization rates were substantially lower than anticipated based on the original iCPR trial (68%), with calculator completion rates of 17% at Health System A and 5% at Health System B. These results were the impetus for the additional site visits and post-deployment provider group interviews described below. At 12 months, after additional refinements based on user feedback at 6 months, utilization rates remained similarly low at 14% and 4% respectively.

### Post-deployment user feedback

Post-deployment, provider-group interviews with over 75 providers in 14 intervention clinics across sites highlighted several persistent usability issues and other potential drivers of the relatively low utilization rates of iCPR2 (Table 3), which were previously unidentified in pre-deployment usability testing. These issues/drivers include: lack of training, perceived increased burden not identified prior to deployment (e.g., too many “clicks,” lack of specificity due to seasonal variation), concerns related to alert fatigue, workflow barriers to use, and lack of provider buy-in with regard to the tool’s utility.

**Table 3.** Examples of post-deployment user feedback and related adapted integrated clinical prediction rule 1 tool modifications per usability theme.

Usability issue/driver	User feedback examples	Modification
Lack of training	Providers that did not receive original academic detailing never tried tool.	Additional academic detailing
Alert fatigue/lack of sensitivity	Lack of specificity in symptom (e.g., cough) yields lack of specificity with regard to firing of tool.	None identified
Workflow barriers	Triggered too early in the process, especially if patient had multiple issues (unaware they could retrieve tool).	Methods to return to tools covered in repeat academic detailing
Added burden	Tool is “one more thing” to do during visits.	Click counts examined, further reductions deemed not possible
Lack of buy-in/tool not useful	Providers familiar with criteria so stopped using/tool did not change how care was provided. After using it providers are comfortable with the criteria so do not need to use it.	None identified

To address the potential for lack of timely training for new providers as a potential driver of low utilization rates, additional academic detailing was held for all physicians and specifically newly on-boarded physicians 6 months post-deployment. An analysis of click counts required for use of Health System A’s calculator and order set for each condition was performed to explore opportunities to relieve burden by reducing clicks (Table 4).

**Table 4.** Adapted integrated clinical prediction rule 1 click counts to calculator and order set per tool version, Health System A.

**Sore throat adult tool**	**Clicks**
To find calculator	1
To complete calculator	4
To find order set	2
**Sore throat child tool**	**Clicks**
To find calculator	1
To complete calculator	4
To find order set	2
**Cough tool**	**Clicks**
To find calculator	1
To complete calculator	3
To find order set	2

As indicated in Table 4, the click breakdown for finding and completing the calculator for each condition, the number of clicks required is minimal. The research team determined that the tool was optimal from this perspective and further reduction in clicks was not feasible. Post-deployment usability findings highlighted other key usability issues and drivers less amenable to quick iteration, yet these findings offer interesting insights into potential limitations and persistent challenges with CDS generally.

## Discussion

This study sought to adapt an innovative CDS tool across diverse ambulatory settings using a user-centered, agile approach to design and implementation. Our user-centered approach allowed for a rapid yet rigorous tool-adaption plan involving substantial engagements with study providers in multiple settings, from pre-deployment, think-aloud sessions to post-deployment group interviews with users. Based on pre- and post-deployment user feedback, the iCPR2 tool was modified iteratively and site specifically and, ultimately, most of the original iCPR CDS tool and its underlying evidence was conserved, while accommodating new workflows in the adapted iCPR2 model.

We hypothesized that the integration of a well-validated, highly usable CDS tool into the EHR would lead to relatively high adoption rates and, as a result, reduced antibiotic prescription and diagnostic test ordering as in the original iCPR study. However, our findings demonstrate the complexity of CDS tools and the likelihood that, even with the use of this state-of-the-art approach to digital health design and testing, there can be persistent external factors impacting adoption rates that we could not address. Process measures from the intervention arm indicated substantially lower utilization rates than hypothesized at the study outset despite our rigorous adaptive design process.

Adoption rates appear to demonstrate a stepped pattern. Essentially, the lower the adoption rate, the more divergent it is from the original iCPR tool design. As indicated in Figure 5, the rate of completion of the iCPR calculator at 6 months post-deployment was 68% in the original iCPR study, but only 14% at the Health System A and 4% in the Health System B affiliated clinics. The stepwise adoption rates suggest there are powerful mediating factors influencing provider utilization of the tools. The ameliorating modifications identified from post-deployment user feedback were primarily technical and not possible in the current EHR environment. In addition, we discovered that adaptive design, with its focus on responsiveness to the end user, led to a tool in the case of Health System B that may have been “overdesigned” to the wishes of the clinicians that the resulting iteration featured almost non-existent visual cues. Resulting cues ended up being so passive that they were ultimately too easy to ignore, diffusing the impact on provider adoption. Although our study was not designed to definitively determine which of these design issues wielded the most influence, our findings (including ongoing feedback from users and key stakeholders) suggest several promising theories.

Key user feedback throughout the iCPR2 tool development and iteration cycle thus far has highlighted alert fatigue and the potential for the tool to contribute to already heavy demands on provider attention as barriers to acceptance. The original iCPR trial was conducted early after the implementation of the EHR at that health system, a time when CDS tools were less pervasive. At the time of the original iCPR study, just a few CDS tools and alerts were implemented within its EHR, and the providers had likely not yet built up significant “alert fatigue.”^31,32^ Since that time, the EHR has become inundated with alerts vying for providers’ attention. It is certainly plausible that the reduction in adoption seen in iCPR2 is in part due to the higher level of CDS alert fatigue creating a higher threshold of importance required for providers to engage with a tool like iCPR2. Unfortunately, the design changes that were recommended for decreasing less clinically relevant triggers were not possible in the current EHR environment; this finding points to the urgency of identifying methods for “smarter” alerting whether through machine learning, personalization by role, or exploring potential to build and incorporate CDS tools outside of the EHR.

Changes in study setting related to professional level of the pool of users (eg, resident versus attending at sites may be an additional issue not identified in feedback contributing to the changes in tool adoption from iCPR1 to iCPR2. The original iCPR study was conducted at a single urban academic hospital clinic composed of a high percentage of trainees compared to attending-level providers. In contrast, iCPR2 was conducted across two large health systems that encompass over 30 distinct primary care sites composed of mostly attending-level physicians. Results from post-deployment key user feedback are similar to other reports documenting differences in CDS engagement among different levels of provider training and career phases.^33^ Should the professional level of the user be a significant factor in CDS usage, the lower concentration of trainees at iCPR2 study sites could be a driver of the lower rates of tool usage compared to the original study.

Provider professional level and level of experience with CDS is an additional human factor to be considered in the UCD process for CDS. The drop-off in CDS adoption among more experienced clinicians corresponds to their qualitative feedback, indicating a belief that evidence provided by the CDS tool became less useful as they became more comfortable with the decision rules. This feedback, together with the fact that the sites in iCPR2 had a more experienced pool of users, suggests there may be value in exploring a more dynamic CDS system able to provide stricter “guardrails” for engagement among inexperienced clinicians (or at least inexperienced with this particular CDS), but less proactive engagement as clinicians gain experience with the tool and their practice behaviors maintain acceptable clinical standards.^33^ Strategies for delineating the specific ways in which experience level impacts adoption of CDS tools as well as potential ways, such as the incorporation of machine learning in this context, are being explored by our team. Final analysis of data from iCPR2 will offer further insight into the role of experience level of provider behavior to inform the development of the next generation of CDS tools.

Low adoption rates may also be an unintentional impact of over-design of the tool to accommodate clinical workflow. A purposeful decision to guard against burden, the user-centered approach may have, particularly in the case of Health System B, allowed the workflow to overcorrect by completely sacrificing visibility for the sake of accommodating expressed provider preference for low burden, passive alerts. This fact limited the tool’s ability to influence provider behavior and engagement with the CDS tool. We are revising Health System B’s workflow to more closely align with Health System A and the original iCPR workflow to understand if the low use reflects design or other factors such as provider culture. This speculation does not suggest that user-centered design is inappropriate for the adaptation of CDS tools in established EHR workflows. Instead, it calls attention to the need for a user-centered design approach in the context of CDS, and potentially other healthcare-related technologies, to balance the priorities of minimizing disruption to workflow and user burden with an understanding that some level of workflow disruption may be required to achieve sufficient user engagement with new tools.

### Limitations

This study is limited by some key challenges. Although we can make some alterations to workflows regarding CDS, we cannot change the actual content of the tool as clinical decision support’s primary role is to provide evidence-based content to users. Additionally, the user interface of the CDS tool is constrained by the EHR vendor software, which limits the design changes able to be made.

Although our research indicates users agree the tools are clinically relevant, they may not agree there is a clinical need for these tools. To the degree to which provider buy-in may be associated with level of training, results from iCPR1 and comparing overall adoption rates in iCPR1 versus iCPR2 (in which study sites contain more experienced providers), suggest that buy-in may be a driver of adoption. However, the design of this study does not provide a way to determine the degree to which buy-in affects adoption rates. Similarly, we cannot adjust for changes in practice of CDS and that providers’ experience with or attitude toward the iCPR2 tool may be shaped by experiences with CDS tools, including “alert fatigue.” A greater breadth of feedback from live usability sessions may have been possible with more than the three providers/six encounters studied; given the resource-intensive nature of this type of data collection and analysis, however, this is a reasonable sample size for live usability studies.

Another limitation may be our choice of common patient symptoms of cough and sore throat for triggers. Not only are more of the triggers not applicable to the clinical situation contributing to increased alert fatigue, but as evaluation and treatment of these common symptoms occurs so often during primary care medical training and practice, more senior residents and attending physicians will have already developed a routine of history, physical exam, and treatment methods. Because they “know what to do,” changing clinical behavior by adding the iCPR2 tools may be less effective. Lastly, our slow response to poor adoption rates posed an additional limitation, born of the fact that unlike a “start-up,” we must work within parameters of a large healthcare organization, which requires great preparation and evidence to implement change.

## Conclusion

This study provides an example of how to leverage the principles and techniques of UCD and HCI approaches to tailor CDS interventions to site-specific workflows to support utilization rates necessary to positively impact clinical outcomes, such as reducing rates of inappropriate antibiotic prescription in the case of iCPR. Although there are limitations to CDS as illustrated above, the adaptability of these tools, when designed and implemented within user-centered frameworks, contributes to CDS tools that are more likely to fit within site-specific workflows and, subsequently, be used more often. We found that although the tools implemented in iCPR2 were designed to meet the specific needs of sites’ workflows and provider preferences as in iCPR1, utilization did not reflect the rates expected from the implementation a similar user-centered process. Our findings offer evidence that although necessary, user-centered design approaches are not sufficient for effective CDS design; our results suggest that additional factors, including structural ones such as the EHR environment and predominance of alert fatigue, are driving the degree to which a CDS tool such as iCPR2 is used. Novel questions and approaches are needed to explore the potential and limitations of UCD approaches to positively impact CDS. Our results suggest examining CDS adoption more broadly to ask the question: how might we address the problem of poor EHR usability more broadly and examine how that might affect adoption of clinical prediction rules such as iCPR2? Additionally, given the digital demands of physicians, exploring adaption of the iCPR2 tool for use by those other clinical roles such as nurses may provide insight into the factors impacting CDS adoption.
